# Acute respiratory failure in COVID-19: is it “typical” ARDS?

**DOI:** 10.1186/s13054-020-02911-9

**Published:** 2020-05-06

**Authors:** Xu Li, Xiaochun Ma

**Affiliations:** Department of Critical Care Medicine, the First Affiliated Hospital, China Medical University, North Nanjing Street 155, Shenyang, 110001 Liaoning Province People’s Republic of China

**Keywords:** Coronavirus, COVID-19, Acute respiratory distress syndrome, Berlin criteria

## Abstract

In December 2019, an outbreak of coronavirus disease 2019 (COVID-19) was identified in Wuhan, China. The World Health Organization (WHO) declared this outbreak a significant threat to international health. COVID-19 is highly infectious and can lead to fatal comorbidities especially acute respiratory distress syndrome (ARDS). Thus, fully understanding the characteristics of COVID-19-related ARDS is conducive to early identification and precise treatment. We aimed to describe the characteristics of COVID-19-related ARDS and to elucidate the differences from ARDS caused by other factors. COVID-19 mainly affected the respiratory system with minor damage to other organs. Injury to the alveolar epithelial cells was the main cause of COVID-19-related ARDS, and endothelial cells were less damaged with therefore less exudation. The clinical manifestations were relatively mild in some COVID-19 patients, which was inconsistent with the severity of laboratory and imaging findings. The onset time of COVID-19-related ARDS was 8–12 days, which was inconsistent with ARDS Berlin criteria, which defined a 1-week onset limit. Some of these patients might have a relatively normal lung compliance. The severity was redefined into three stages according to its specificity: mild, mild-moderate, and moderate-severe. HFNO can be safe in COVID-19-related ARDS patients, even in some moderate-severe patients. The more likely cause of death is severe respiratory failure. Thus, the timing of invasive mechanical ventilation is very important. The effects of corticosteroids in COVID-19-related ARDS patients were uncertain. We hope to help improve the prognosis of severe cases and reduce the mortality.

## Introduction

**Fig. 1:**
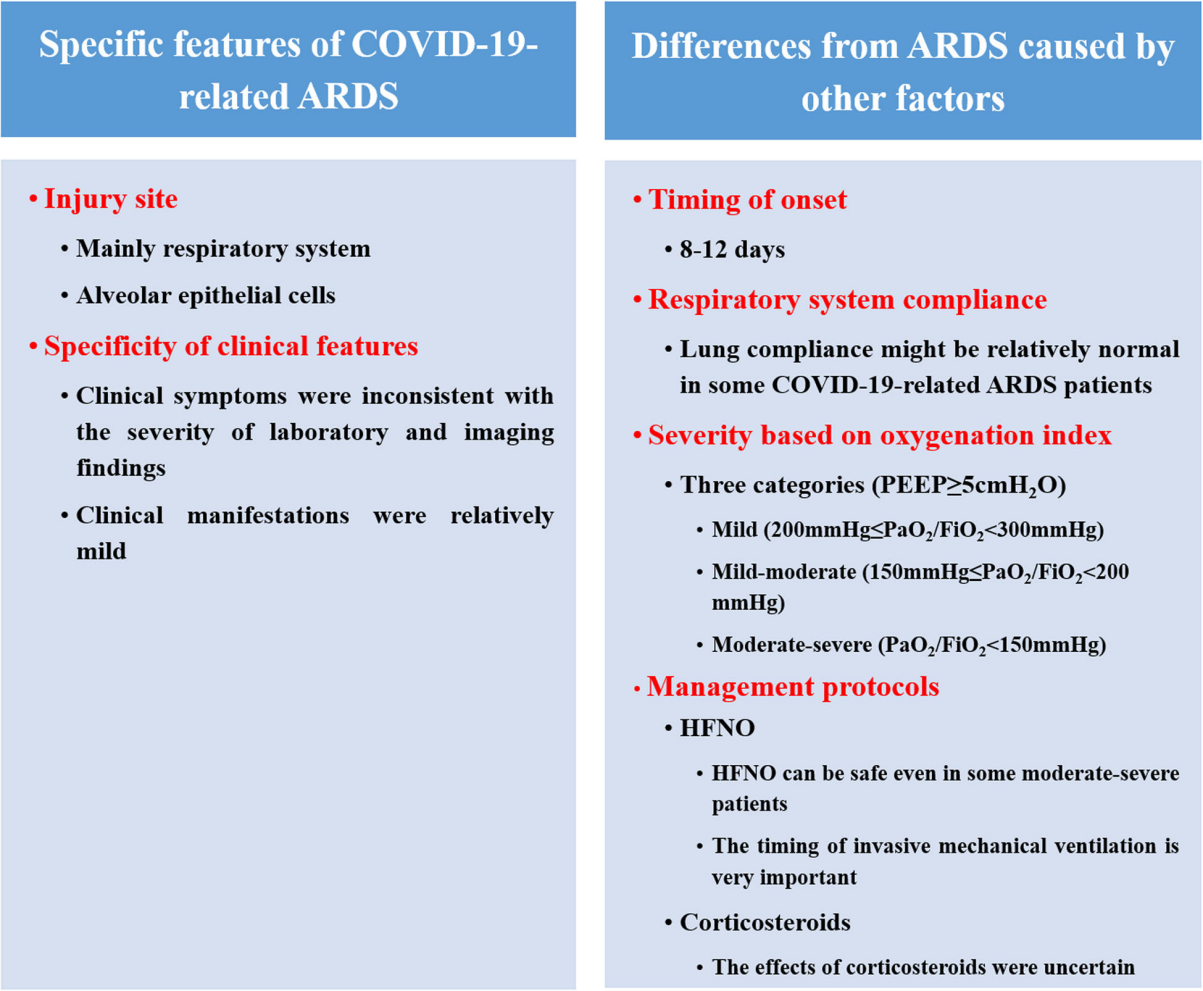
Summary of characteristics of COVID-19-related ARDS

In December 2019, an outbreak of coronavirus disease 2019 (COVID-19), which was caused by severe acute respiratory syndrome coronavirus 2 (SARS-CoV-2), broke out in Wuhan, China [1–3]. The World Health Organization (WHO) declared it a significant threat to international health [4]. COVID-19 was of clustering onset and mainly affected the respiratory system with some patients rapidly progressing to acute respiratory distress syndrome (ARDS); other organ functions were less involved [5, 6]. These patients were likely to be admitted to the intensive care unit (ICU) and might die. The elderly and those with comorbidities are at highest risk of death. The death appeared to be related to ARDS [7]. Although several studies have reported the clinical features of COVID-19 [1, 8–13], our understanding about it remains limited [14]. Can we consider all the cases of acute respiratory failure associated with COVID-19 as ARDS? The answer is probably no. Based on current reports and our experience in the management of COVID-19-related ARDS patients, we realized that there are many differences between COVID-19-related ARDS and ARDS caused by other factors as defined by Berlin criteria, and therefore differences in treatment. Thus, we aimed to describe the characteristics of COVID-19-related ARDS and to elucidate the differences (Fig. 1).

## Specific features of COVID-19-related ARDS

### Injury site of COVID-19

ARDS occurs as a result of an acute systemic inflammatory response, which can be caused by insults to the lung, either direct or indirect. The early exudative stage presents diffuse alveolar damage with destruction of epithelial and endothelial cells. COVID-19 mainly affected the respiratory system with minor damage to other organs. Studies reported that acute myocardial injury (7.2–17%) and acute renal injury (2.9–15%) could occur in severe patients. The reported incidence of ARDS was 15.6–31%, higher than that of other organ injuries [1, 8–11].

The most common respiratory symptom of COVID-19 is dry cough (59.4–82%) [1, 8–11]. Sputum production was less. It suggested that injury to the alveolar epithelial cells was the main cause of COVID-19-related ARDS, and endothelial cells were less damaged with therefore less exudation. Endothelial cells line the inner surface of blood vessels in all organs. It was possible that due to less damage to the endothelial cells, other organ functions were less involved in COVID-19 patients.

### Specificity of clinical features

The respiratory system was mainly involved in COVID-19 patients as mentioned above. Some patients had a low oxygenation index, indicating severe respiratory failure. Chest imaging findings suggested the involvement of both lungs. Chest computed tomography (CT) scans usually showed multifocal bilateral patchy shadows and/or ground-glass opacities; some patients showed a mixed pattern of ground-glass opacities and consolidation [15]. The CT results indicated diffuse and severe lung injury. However, the clinical manifestations were relatively mild in some patients. These patients might have no complaint of dyspnea, no significant increase in respiratory rate, and no respiratory distress. Hemodynamics and indexes of tissue perfusion such as lactate were also relatively stable. The clinical symptoms were inconsistent with the severity of laboratory and imaging findings. However, these patients may deteriorate rapidly and need to be monitored closely. Blood carbon dioxide levels may be a meaningful indicator for invasive mechanical ventilation.

## Differences from ARDS caused by other factors

### Timing of onset

The ARDS Berlin criteria defined that for a patient to be diagnosed as having ARDS, the onset must be within 1 week of a known clinical insult or new or worsening respiratory symptoms [16]. The reported onset of COVID-19-related ARDS was similar in different studies. Huang et al. [1] first reported 41 cases of COVID-19 in which the median time from onset of symptoms to ARDS was 9.0 days (8.0–14.0). Subsequently, Wang et al. [9] reported 138 cases of COVID-19 in which the median time from the first symptom to ARDS was 8.0 days (6.0–12.0). Zhou et al. [11] reported the median time from illness onset to ARDS was 12.0 days (8.0–15.0). Studies by Chen et al. [8] and Guan et al. [10] did not report the onset of ARDS. As the onset time of COVID-19-related ARDS was 8–12 days, it suggested that the 1-week onset limit defined by ARDS Berlin criteria did not apply to COVID-19-related ARDS. It reminded us to pay more attention to the development of ARDS in patients with the course of more than a week, so as to treat timely.

### Respiratory system compliance

Not all the cases of acute respiratory failure caused by COVID-19 were ARDS. The typical CT findings of COVID-19 showed bilateral ground-glass shadow with a peripheral lung distribution [15]. Although there was consolidation and exudation, it was not a “typical” ARDS image. ARDS is a condition associated with many disease processes, resulting in reduced lung compliance and severe hypoxemia [16]. Lung compliance might be relatively normal in some COVID-19-related ARDS patients who met ARDS Berlin criteria. This was obviously inconsistent with ARDS caused by other factors. In addition, the lung compliance was relatively high in some COVID-19-related ARDS patients, which was inconsistent with the severity of hypoxemia.

### Severity based on oxygenation index

According to the Berlin definition, ARDS is divided into three stages based on oxygenation index (PaO_2_/FiO_2_) on positive end-expiratory pressure (PEEP) ≥ 5 cmH_2_O: mild (200 mmHg < PaO_2_/FiO_2_ ≤ 300 mmHg), moderate (100 mmHg < PaO_2_/FiO_2_ ≤ 200 mmHg), and severe (PaO_2_/FiO_2_ ≤ 100 mmHg) [16]. ARDS classification determines both the severity of the disease and choice of treatment protocol. A previous study reported that more than 50% of patients with moderate and severe ARDS according to the Berlin definition did not show diffuse alveolar damage [17]. In fact, the specific ranges under which the hypoxemia is evaluated differ among clinicians. From the perspective of therapy, the intensity of adjunctive treatment varies according to the degree of hypoxemia. Thus, we need a more suitable classification of ARDS severity that can accurately identify patients for a specified therapy.

Till now, the clinical features of COVID-19-related ARDS are still unclear. There are no specific monitoring and implementation protocols. Under the current situation, a number of designated hospitals and dozens of medical teams from different provinces have participated in the treatment. The unified ARDS treatment standard is needed in order to improve the uniformity and thus reduce the mortality. Therefore, experts from the national health commission of China developed a standard treatment protocol for COVID-19 based on their experience. COVID-19-related ARDS was divided into three categories based on oxygenation index (PaO_2_/FiO_2_) on PEEP ≥ 5 cmH_2_O: mild (200 mmHg ≤ PaO_2_/FiO_2_ < 300 mmHg), mild-moderate (150 mmHg ≤ PaO_2_/FiO_2_ < 200 mmHg), and moderate-severe (PaO_2_/FiO_2_ < 150 mmHg) [18]. The new stratification for COVID-19-related ARDS determines personalized treatment for different patients. In fact, a number of ARDS treatments, including prone positioning and neuromuscular blockers, are recommended for patients with PaO_2_/FiO_2_ less than 150 mmHg. This indicates that Berlin classification is not suitable to define the severity of ARDS and accurately guide the corresponding treatments.

### Management protocols

#### High-flow nasal oxygen in COVID-19-related ARDS patients

Hypoxemic respiratory failure in ARDS generally results from intrapulmonary ventilation-perfusion mismatch or shunt and usually requires mechanical ventilation. Compared to standard oxygen therapy, high-flow nasal oxygen (HFNO) reduces the need for endotracheal intubation in ARDS patients [19]. WHO recommended that HFNO should only be used in selected patients with hypoxemic respiratory failure [20]. Studies indicated that HFNO is more suitable for patients with mild ARDS. However, according to clinical situations, HFNO can be safe in both mild and mild-moderate COVID-related ARDS patients, and even some moderate-severe patients. Some patients with an oxygenation index of 100 mmHg can remain relatively stable with the support of HFNO. This is clearly inconsistent with the stratified treatment strategies of ARDS caused by other factors.

Although COVID-19 may be associated with myocardial injury and arrhythmia as reported [8, 9, 11], there is currently no evidence that myocarditis is the cause of death. The respiratory system is the most commonly involved for COVID-19, and some cases can rapidly progress to ARDS, which requires venous-venous extracorporeal membrane oxygenation (V-V ECMO) in the most severe cases. To date, no patients with severe arrhythmia or acute heart failure due to acute myocarditis have been reported to require venous-arterial ECMO (V-A ECMO) treatment. Cardiac injury was diagnosed by elevation of cardiac biomarkers in serum or new abnormalities in electrocardiography and echocardiography. However, serum lactate dehydrogenase (LDH) and creatine kinase-MB were elevated more commonly than hypersensitive troponin I in COVID-19 patients as reported [1, 9]. The pathological findings of a COVID-19-related ARDS patient by Xu et al. [21] indicated that there were no obvious histological changes seen in heart tissue. Therefore, the diagnosis of acute myocardial injury needs further consideration. Thus, the more likely cause of death is severe respiratory failure. Therefore, the timing of invasive mechanical ventilation is very important. Since severe COVID-19 patients may deteriorate rapidly, patients receiving HFNO should be closely monitored and cared for by experienced personnel capable of endotracheal intubation at any time. Currently published studies did not report the proportion of different respiratory support according to COVID-19-related ARDS classification. Further research is expected to provide more evidence for the use of HFNO in COVID-19-related ARDS patients.

#### Corticosteroids in COVID-19-related ARDS patients

Corticosteroids are considered a potential treatment for ARDS because of their role in reducing inflammation and fibrosis. Although the use of corticosteroids in ARDS patients remains controversial, treatment with corticosteroids is currently the only pharmacological intervention that may reduce morbidity and mortality. It is reported that treatment with high-dose corticosteroids for a prolonged period of time could accelerate the improvement of ARDS [22]. Furthermore, methylprednisolone shortened periods of need for invasive mechanical ventilation and lowered mortality in ARDS patients [23]. However, WHO recommended that systemic corticosteroids should not be routinely administered to COVID-19 or COVID-19-related ARDS patients [20]. Studies reported that less than half of the COVID-19 patients were given systemic corticosteroids, mostly in severely ill patients with ARDS [1, 9, 11]. Low-to-moderate dosage was administered depending on the severity of the disease, for as short time of treatment as possible. Although it has been reported that treatment with methylprednisolone may be beneficial for COVID-19-related ARDS patients [24], the effect of corticosteroids in such patients is still uncertain and needs to be further evaluated. In particular, the use of corticosteroids may affect virus clearance in COVID-19 patients. Further evidence is needed to evaluate the role of systemic corticosteroid therapy and its impact on long-term prognosis in this group of patients.

## Conclusion

COVID-19 is highly infectious and can lead to fatal comorbidities especially ARDS. There are currently no recommended specific anti-COVID-19 treatments, so supportive treatment is important. Fully understanding the characteristics of COVID-19-related ARDS is conducive to early identification and precise treatment. We hope to help improve the prognosis of severe cases and reduce the mortality.

## Data Availability

Not applicable.

## Abbreviations

ARDS: Acute respiratory distress syndrome
COVID-19: Coronavirus disease 2019
CT: Computed tomography
HFNO: High-flow nasal oxygen
ICU: Intensive care unit
LDH: Lactate dehydrogenase
PEEP: Positive end-expiratory pressure
SARS-CoV-2: Severe acute respiratory syndrome coronavirus 2
V-A ECMO: Venous-arterial extracorporeal membrane oxygenation
V-V ECMO: Venous-venous extracorporeal membrane oxygenation
WHO: World Health Organization
